# Multi-casting approach for vascular networks in cellularized hydrogels

**DOI:** 10.1098/rsif.2016.0768

**Published:** 2016-12

**Authors:** Alexander W. Justin, Roger A. Brooks, Athina E. Markaki

**Affiliations:** 1 Department of Engineering, University of Cambridge, Cambridge CB2 1PZ, UK; 2 Division of Trauma and Orthopaedic Surgery, Addenbrooke's Hospital, Cambridge CB2 0QQ, UK

**Keywords:** vascularization, three-dimensional printing, vascular networks, hydrogel

## Abstract

Vascularization is essential for living tissue and remains a major challenge in the field of tissue engineering. A lack of a perfusable channel network within a large and densely populated tissue engineered construct leads to necrotic core formation, preventing fabrication of functional tissues and organs. We report a new method for producing a hierarchical, three-dimensional (3D) and perfusable vasculature in a large, cellularized fibrin hydrogel. Bifurcating channels, varying in size from 1 mm to 200–250 µm, are formed using a novel process in which we convert a 3D printed thermoplastic material into a gelatin network template, by way of an intermediate alginate hydrogel. This enables a CAD-based model design, which is highly customizable, reproducible, and which can yield highly complex architectures, to be made into a removable material, which can be used in cellular environments. Our approach yields constructs with a uniform and high density of cells in the bulk, made from bioactive collagen and fibrin hydrogels. Using standard cell staining and immuno-histochemistry techniques, we showed good cell seeding and the presence of tight junctions between channel endothelial cells, and high cell viability and cell spreading in the bulk hydrogel.

## 1. Introduction

A perfusable vascular network is necessary to support the mass transport requirements of a metabolically active and highly populated tissue. The lack of a three-dimensional (3D) system has held back progress into fabrication of complex tissues and organs, as diffusion from the surface of a tissue construct becomes unfeasible for rapid delivery of oxygen and nutrients [1]. A functioning blood vessel network, which is 3D, hierarchical and perfusable, is thus a necessary requirement of most functional tissues; the use of channel hierarchy enabling both extensive coverage of the tissue while simultaneously providing the low pressure heads required for efficient diffusion in and out of the bulk. Porous materials, while improving the permeability of tissue engineered constructs, are still limited in the capacity for this exchange in thick samples especially in scaffolds containing biologically relevant cell densities [2]. Additionally, blood vessel infiltration by the host's vasculature tends to be a slow process, meaning limited functionality of implanted scaffolds [3]. Thus tissue engineering, which seeks to fabricate new tissues and organs, requires approaches for the formation of 3D vascular networks in materials suitable for supporting tissue development, of which hydrogels are the most important group.

Current vascularization methods are limited in their capacity for supporting a large volume and achieving full three-dimensionality; a variety of techniques have been suggested for forming vascular systems, each with specific limitations. Layer-by-layer approaches, such as those based on lithography, have been reported [4–11] but are limited by the lack of three-dimensionality, which is achievable from a single plane of channels. However, such approaches do show that a perfusable vascular system greatly improves the functionality of a tissue construct and can display characteristics of an active vascular system, such as angiogenic sprouting from the channels into a bulk hydrogel [4]. Gelatin and alginate have previously been used as sacrificial materials for the formation of planar vascular networks, in which polydimethylsiloxane (PDMS) moulded casts of hydrogel templates yielded perfusable networks at very fine length-scales [9,12].

A range of direct 3D printing-based approaches have created vascular networks in three-dimensions [13]. However, they often require the casting material to be chemically or photo-cross-linked, requiring cells to be seeded after casting which leads to a lack of cell uniformity in the bulk of the tissue construct, or they use materials that are synthetic in origin and thus often have properties that limit cellular activities [14–16]. One promising sacrificial 3D printing technique uses 3D filament networks of carbohydrate glass, allowing casting in ideal tissue engineering materials, and which generates open channels upon immersion in water [17]. However, there are issues with the cytotoxicity from the dissolution of carbohydrate glass templates (osmotic damage to cells) and to prevent early degradation, the template requires a synthetic coating (PLGA) [3]. Another promising technique uses printing of Pluronic F127 as the sacrificial material [18–21], which can be printed alongside cell-loaded hydrogel materials.

The direct printing of these sacrificial materials limits the complexity and structure of the vascular network that is producible, due to certain properties of the materials (e.g. hydrophilicity, gelling speed, removal method). For instance, in the case of agarose [3] or carbohydrate glass printing [17], a sequence of one-dimensional (1D) filaments is extruded out of the printhead which touch at various locations to produce network junctions. In the case of the carbohydrate glass, the thickness of the channel is determined by the speed of the printhead, limiting the structure of the finer features that can be fabricated [17].

Other work has used thermoplastic 3D printed models to produce internal channels in porous scaffolds, such as with freeze-dried collagen [22,23] and PCL (collagen coating on the channels) [24]. However, they require freezing and harsh solvents as a means of formation of the scaffold and removal of the channel template material. Thus, they must remain acellular until after the process is completed and both approaches require the collagen to be chemically cross-linked for stability. Finally, 3D printed materials have recently been cast in PDMS and removed to reveal a 1D helical channel (500 µm in diameter) [25]. The use of PDMS as the bulk material limits the use of cells to the channels only.

We have developed a novel process in which standard 3D printed thermoplastic models, which are highly customizable and reproducible, are converted into a gelatin hydrogel material. This can then be used as a vascular network template in highly bioactive hydrogel materials, which are pre-loaded with cells. The ability to use 3D printed materials in this way is achieved by casting an alginate intermediary, which is ionically cross-linked with calcium, around the 3D printed model. Alginate hydrogels are stable at high temperatures (approx. 100°C), and do not decay in acetone, both of which are required to remove the thermoplastic (polyester-based) model in its entirety. By making use of the different physical and chemical properties of the 3D printed material, and of alginate and gelatin, we are able to selectively add and remove positive and negative casts of the original network design, resulting in a channelled construct made from thermo-gelled collagen or enzymatically cross-linked fibrin hydrogel. The use of gelatin gel as a vascular template has the added advantage of enabling prompt perfusion once the final construct has formed.

Recently, a 3D printed material has been converted into an alginate template material and used in agarose and genipin-cross-linked gelatin constructs, with trifurcating channel features down to 400 µm, supporting liver cancer cells [26]. In our study, via a different set of materials, we produced a bifurcating template, which was hierarchical in nature with features down to 200 µm. The template material was removable within 20 min of casting, meaning minimal delay prior to perfusion, which can be performed at physiologically comparable rates. A flow rate of approximately 2 ml h^–1^ through the 200 µm channels can be achieved by applying 32 ml h^–1^ at the 1 mm inlet [17]. The implantable, bioactive hydrogel materials collagen [27], as the major component of extracellular matrix, and fibrin [28], as the major protein precursor for wound healing, were used as our final constructs. These materials supported primary cells; here, we used endothelial cells in the channels and connective tissue cells, which showed spreading in the hydrogel constructs.

## 2. Material and methods

### 2.1. Cell culture

Human umbilical vein endothelial cells (HUVEC, Public Health England) and human dermal adult fibroblasts (HDFs, Public Health England) were used for the experiments. The cells were trypsinized using TrypLE Express (Life Technologies), centrifuged at 250 g for 5 min, and counted using a Scepter Cell Counter (Millipore). HUVECs were cultured in a T75 flask to 80% confluence, using endothelial growth medium (no vascular endothelial growth factor or platelet-derived growth factor) (EGM Plus, Lonza), and HDFs were cultured in a T75 flask to 80% confluence using high-glucose Dulbecco's modified Eagle's medium (Life Technologies) containing 10% fetal bovine serum (FBS, Life Technologies).

HUVECs were imaged using Green Cell Tracker dye (Life Technologies), allowing for immediate imaging and tracking of cell seeding and distribution in the channels. Lyophilized fluorescent dye was resuspended in dimethyl sulfoxide (DMSO, Sigma) at 10 mM and stored at −20°C until use. A working solution was prepared in DMEM at 10 µM. Plated cells were washed with phosphate-buffered saline (PBS, Life Technologies) and the working solution added. Flasks were incubated at 37°C for 45 min prior to trypsinization.

### 2.2. Three-dimensionally printed design and printing

**Figure 1.**
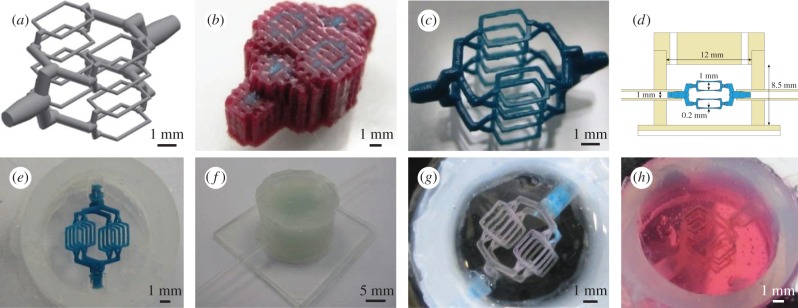
Process steps for converting CAD model into hydrogel channels. (*a*) CAD design, consisting of 1 mm inlet symmetrically bifurcating to sixteen 200–250 µm channel features. Each of the finest channels is 2 mm in length. (*b*) Using a Solidscape 3D printer, models are produced in support wax. (*c*) Support wax is removed using a selective solvent to reveal a high precision thermoplastic model. (*d*) Schematic of custom-made silicone chamber; consisting of a silicone sheet mounted on a glass imaging slide, thick-walled silicone tubing for chamber walls, flexible silicone tubing for inlets, and a CNC-milled silicone lid, which has two holes for casting. Also shown is the 3D printed model from (*a*) for size comparison. (*e*) Model is mounted into silicone chamber. (*f*) Chamber is filled with alginate hydrogel and a lid is fitted to the chamber. (*g*) Following model removal with heat and acetone, gelatin is infiltrated into the channels, and the alginate is removed by chelation. (*h*) Gelatin template is cast in collagen (shown here) or fibrin hydrogel, which can be loaded with cells prior to gelation.

The CAD model vasculature was designed using Autodesk Inventor 2014 (figure 1*a*); the structure consists of a 1 mm inlet which symmetrically bifurcates into a series of 200–250 µm channels, which then converge into a single outlet of 1 mm. Thermoplastic models in support wax were a gift from Solidscape UK. Models were fabricated using 12 µm layer thickness and 10 base support layers, on a 3Z Studio printer. Following printing, models in support wax were removed from the ceramic base (8 mm) by placement on a hot plate at 120°C for 7 min. Models in support wax could then be gently removed using stiff card. To remove the support wax from the models (figure 1*b*), they were put in a bath of selective solvent (BioAct VSO, Solidscape) and placed in an oven at 55°C for 4 h. The model was subsequently stored in fresh solvent at room temperature, until use (figure 1*c*).

### 2.3. Silicone chamber design and model mounting

The vascularized hydrogel was produced in a custom-made silicone chamber. For the base, a 1 mm silicone sheet (Silex) was adhered to a glass imaging slide (Fisher Scientific) and a section of 12 mm internal diameter silicone tubing (VWR) attached to this sheet, both using silicone sealant (Dow Corning). Two holes were drilled on opposite sides of the chamber and flexible silicone tubing with 1 mm internal diameter (VWR) fitted. A silicone lid was made using a CNC milling machine from a 3 mm silicone sheet (Silex), into which two 2 mm filling holes were drilled (chamber design shown in figure 1*d*). The models were briefly dried on tissue paper and then very carefully placed between the filled silicone inlets using tweezers. The inlets were rotated so as to angle the model such that all channel features are visible under an optical microscope at the same time (figure 1*e*). The silicone inlets were then super-glued in place.

### 2.4. Alginate preparation

Calcium alginate gels were prepared using an ‘internal setting’ method. This involved adding 300 mM CaHPO_4_ (Sigma) and 600 mM gluconolactone (Sigma) to 7.5% (w/v) sodium alginate (Sigma). In order to prevent the trapping of bubbles between the fine features of the 3D printed template, the chamber was first filled with DI water. The viscous alginate precursor solution was then slowly injected into the silicone chamber, displacing the water and fully encapsulating the thermoplastic model. The gluconolactone functions as an acidifier and causes the slow release of calcium, enabling a controlled gelling mechanism and a uniformly cross-linked hydrogel. The alginate gel was left overnight at 4°C for maturation (figure 1*f*).

### 2.5. Model removal and gelatin template preparation

The model material was removed by placing the chamber in a bath of near-boiling DI water. After 5 min, the liquid model material was removed from the channels by gentle suction with a 1 ml syringe. Following 10 min on ice, the channel structure was cleaned by injecting 2 ml of acetone into the channels to purge any remaining model material. Subsequently, 6 ml of 250 mM CaCl_2_ (Sigma) was injected around the network which cross-links the surface of the alginate channels more thoroughly, without causing significant syneresis.

Liquid gelatin (Sigma, porcine skin, low bloom, 15% (w/v), pH 7.4) was infiltrated into the channel structure via suction. The chamber lid was removed and the chamber placed in a bath containing 200 mM sodium citrate (Sigma) and 200 mM glycine (Sigma) to chelate the calcium cross-linking ions, and was then left for 24 h at 4°C, at which point the alginate was fully dissolved into solution. Once the alginate gel had dissolved, the chamber was placed in a bath of sterile, calcium free PBS to remove any residual alginate in the chamber, and a lid attached under liquid, which was then sealed using super-glue. The chambers were finally purged of any remaining citrate or alginate by flowing 5 ml of PBS through the lid holes (figure 1*g*).

### 2.6. Extracellular matrix hydrogel preparation

Collagen hydrogels were used at 7.5 mg ml^–1^ final concentration. Soluble collagen solution was prepared using rat-tail tendon. We solubilized the tendon using 0.1% (v/v) acetic acid on a magnetic stirrer (250 ml g^–1^ tendon) for 60 h at 4°C, and centrifuged the solution at 9000*g* for 90 min. The collagen was subsequently lyophilized, weighed and resuspended at the required stock concentration in 0.1% (v/v) acetic acid. Collagen was gelled using a standard approach [6], employing the addition of 10× M199 (Sigma), 1 M NaOH, and 0.2% (w/v) NaHCO_3_ (Sigma), in order to raise the ionic content and pH of the collagen solution, thereby inducing a gelling response. HDFs were carefully mixed into the neutral precursor solution at 1 × 10^6^ cells ml^−1^, just prior to casting. Owing to the high viscosity of the collagen solution, it was injected slowly on ice into the chamber using a syringe pump at 15 ml h^–1^, displacing the PBS supporting the gelatin template (figure 1*h*). Following 20 min at room temperature, the chamber was placed for an additional 20 min in a sterile 37°C bath, which rapidly gelled the collagen and melted the gelatin.

Fibrin hydrogels were produced using a final concentration of 10–20 mg ml^–1^ bovine fibrinogen (Millipore) and 2 U ml^–1^ human plasma thrombin (Sigma). Lyophilized fibrinogen was resuspended in pre-warmed 0.9% (w/v) NaCl at 60 mg ml^–1^ and thrombin was resuspended in 0.9% (w/v) NaCl at 50 U ml^–1^. Both were stored at −20°C until use. In total, 0.1 M NaOH was added to the fibrinogen solution to raise the pH to 7.4. HDFs, in cell medium, were carefully mixed into the precursor solution at 1 × 10^6^ cells ml^−1^ and thrombin was added just prior to casting. The solution was subsequently loaded into a 1 ml syringe and injected into the chamber via the lid holes, displacing the PBS supporting the gelatin template. Following 5 min at room temperature, the chamber was placed in a sterile 37°C bath for an additional 15 min, which melted the gelatin.

### 2.7. Gelatin removal and channel seeding

Gelatin was removed from the channel network by gentle suction of PBS at 37°C through the inlets. Channels were checked for patency using fluorescent 1 µm red tracer beads (Life Technologies). HUVECs were subsequently suspended in 100 µl of endothelial growth medium and injected into the channels at 2 × 10^7^ cells ml^−1^. The chamber was left for 5 h-overnight prior to commencing perfusion. Non-adherent HUVECs were flushed from the channels and perfusion continued with endothelial growth medium at 0.1 ml h^–1^ for 7 days, using a syringe pump. For HDF experiments, high-glucose DMEM containing 10% FBS was perfused at a range of flow rates (1–30 ml h^–1^ at the 1 mm inlet) for 7 days using a peristaltic pump, and the medium reservoir was replaced every 2 days.

### 2.8. Characterization of microchannel formation

After 7 days of perfusion, the development of tight cell–cell junctions between the channel HUVECs was determined using CD31 immunocytochemistry. Cells were fixed by injecting 4% (w/v) paraformaldehyde (PFA) into the channels and leaving the chamber at 4°C for 1 h. Following three 5 min washes with PBS, a permeabilizing solution containing 0.25% (v/v) Triton X-100 (Sigma) was injected into the channels for 10 min at room temperature, followed by a blocking solution of 1% bovine serum albumin (BSA, Sigma) for 30 min at room temperature. The primary antibody, mouse monoclonal anti-CD31 (HEC7, Abcam), was added at 1 : 100 in 1% BSA, injected through the inlet and incubated overnight at 4°C. Unbound antibody was flushed out using three 5 min washes of PBS, and a secondary antibody, goat anti-mouse IgG H&L conjugated to AlexaFluor 568 (Abcam), was added at 1 : 200 in 1% BSA, injected into the channels, and incubated for 2 h in the dark at room temperature. The unbound antibody was subsequently flushed out using three 5 min washes of PBS. Fluoroshield with 4′,6-diamidino-2-phenylindole (DAPI, Life Technologies) was subsequently injected as a nuclear counterstain. Cell viability was performed using a Live–Dead kit (Life Technologies). Fibrin gels containing 1 × 10^6^ cells ml^−1^ were cross-sectioned through the centre of the construct, and the surface stained with 1 µl ml^–1^ calcein AM and 4 µl ml^–1^ ethidium homodimer-1. After 20 min, slices were washed in PBS and imaged.

### 2.9. Imaging

Imaging was performed using phase contrast and epi-fluorescent microscopy (Zeiss Observer.Z1 with ORCA-Flash4.0). Images were processed using Zen software (Zeiss) and ImageJ. Other images were taken using an overhead microscope (Olympus SZX16 with PixeLINK camera and software), and also with an optical camera (Canon Digital IXUS70).

### 2.10. Statistical analysis

Statistical analysis of live–dead data was performed using IBM SPSS Statistics 23. Cells were manually counted using ImageJ by taking representative sample areas of 1 mm^2^ (at least four were analysed per flow rate). A Levene's test showed variances were not homogeneous and so a comparison of values was carried out using a Kruskal–Wallis and a Games–Howell post hoc test. Differences were considered statistically significant for *p* < 0.05. Data are presented as the mean ± s.d.

## 3. Results and discussion

The channel network produced for this study has a hierarchical structure, with channels bifurcating symmetrically from 1 mm to 200–250 µm, producing a single inlet and outlet, and 16 channels to support a large 3D volume. 3D printed models are prepared using a commercially available printer, mounted into a custom-made silicone chamber, which is filled with alginate gel. Following evacuation of the 3D printed model material from the alginate cast, gelatin is subsequently infiltrated into the channels and the alginate is removed by chelation of the cross-linking calcium ions. This template is then cast in a collagen or fibrin gel, which can be pre-loaded with cells, and the gelatin liquefied at 37°C.

**Figure 2.**
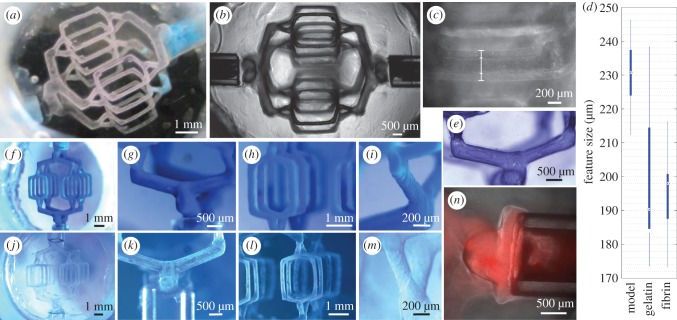
Fidelity of hydrogel channels and gelatin template to 3D printed model design. (*a*) Gelatin template without acetone wash, displaying three-dimensionality of the network. Note that without acetone wash, model material remains on the walls of the template. (*b*) Phase contrast image of gelatin template, displaying minimal variation between individual features of the template. (*c*) Gelatin halo is formed around the desired template features when the alginate hydrogel is insufficiently cross-linked or gelatin infiltration is undertaken at too high a temperature. Line denotes outer boundaries of gelatin halo and templated feature. (*d*) Comparison of the diameters of the templates finest features, at different stages of the fabrication process. The CAD model had finest features of size of 170 µm. Box plot displays minimum and maximum diameters, and 1st and 3rd quartiles. Circles represent median diameters. (*e*) Surface contours of gelatin template display 12 µm thick terraces from layer-by-layer 3D printing process. Correspondence of 3D printed model (*f–i*) to gelatin template (*j–m*) for specific large-scale features. (*n*) Tight interface between fibrin hydrogel and silicone inlet. Shown in red are 1 µm fluorescent beads, which do not leak around the side of the inlets for a 20 mg ml^–1^ fibrin gel.

The use of standard CAD and commercial 3D printing to generate the first stages of the process conveys a high degree of precision and reproducibility to the gelatin (figure 2*a,b*), and thus the vascular channel structure. In particular, Solidscape inkjet technology enables very precise features to be fabricated at significant volumes, and the use of support wax enables a wide range of designs, including overhanging features. Additionally, there is some choice in the shape of the channel cross-section and the morphology of the channel junctions. Thus, our approach enables gelatin templates to be fabricated with a highly complex and fine network architecture, which is fully scalable.

The CAD design in this study was influenced by a number of considerations, including a correspondence to physiological channel structures and fluidic conditions, but also to an ease of handling and imaging. Vascular systems are constrained physiologically by a number of relationships, most notably Murray's law relating parent and daughter branch diameters, usually quoted as the sum of cubic terms which, among other results, yields a constant shear force around the whole network [29,30]. In this study, we have designed a network that uses the sum of squared terms. If we were to follow the sum of cubic terms, a higher number of bifurcations would be needed and the same volume would have a much higher density of channels. Mounting of the 3D printed models requires an inlet size that is not difficult to handle; hence, we used inlets with a diameter of 1 mm. For ease of imaging, a template was desired with fewer channels such that there is minimal overlap between them. The chamber is constrained in this study to a volume of 1 ml in order to maximize cell concentration, though we postulate that much larger volumes are possible as this is a casting-based technique, and thus a closer proximity to Murray's law is possible in the future.

The angles between parent and daughter branches are chosen as 70°, to minimize turbulent flow at junctions while also maximizing the volume supported by the finest channels of the network. The finest channels are 2 mm in length and are separated by 1.5 mm in plane and 1 mm between planes. Thus, these thoroughfare channels enable diffusion of nutrients and oxygen into a significant proportion of the bulk hydrogel.

The choice of the gelatin and alginate used is essential for this process to be successful. A low bloom (80 g) gelatin is used that can withstand alginate removal and subsequent casting with collagen or fibrin, but has a viscosity which is low enough to be easily removed from the final hydrogel. It is type A gelatin, which has a better gel strength to viscosity ratio than type B [31]. A concentration of 15% (w/v) is sufficient for the chelation and casting process, and has a melting point such that a low viscosity liquid is produced upon melting at 37°C. However, the gelatin template still requires mechanical support from the surrounding liquid in order to preserve its structure, prior to casting.

To enable the alginate to be cast and removed effectively, a low viscosity sodium alginate is used at a concentration of 7.5% (w/v), which provides sufficient rigidity for the fabrication process; in particular resisting syneresis from high temperatures, solvent exchange and CaCl_2_ addition. The alginate gels are fabricated using an internal setting process using CaHPO_4_ and gluconolactone as an acidifier, which slowly releases the calcium producing a uniform alginate hydrogel. Overloading the alginate gel with calcium at gelation causes significant syneresis and thus poor reproduction of the original CAD design. However, if extra calcium is not added, the pore size of the alginate gel is sufficiently large to allow the liquid gelatin to penetrate the surface of the alginate gel. Following chelation, this generates a ‘halo’ of gelatin around the templated gelatin channels, greatly increasing the diameter of the finest channels, as illustrated in figure 2*c*. Additionally, the molecular weight of gelatin reduces with increasing temperature [18] and thus using gelatin at higher temperatures produces a thicker halo.

To overcome these issues, one must further cross-link the channel surfaces of the alginate hydrogel by injecting CaCl_2_ via the inlets, and use gelatin at 37°C. Sufficient cross-linking of the alginate at the channel surface prevents the halo effect and produces channels close to the 3D printed model design. In order that the injected calcium penetrates to the alginate surface, the 3D printed model material must be completely removed; a layer of model material prevents the calcium from cross-linking the alginate sufficiently. To this end, the remaining (polyester-based) model material is dissolved using acetone once the channels have become patent. If acetone is not used, the gelatin template is turbid and some model material can be left on the surface (figure 2*a*). In total, 250 mM CaCl_2_ is then injected around the channel network, cross-linking the alginate channel surface but without causing significant syneresis of the whole gel.

The choice of alginate as an intermediary casting medium enables fine features of the network to be translated into the gelatin network, though there is some variation between the CAD design and the 3D printed model, between the model and the gelatin template, and between the gelatin template and hydrogel channels, as shown in figure 2*d*. As the 3D printed models are produced with features at the lower limit of the Solidscape machines, there is some variation in their size; a CAD feature with a diameter of 170 µm yielded a model with features in the range of 200–250 µm.

Using negative pressure, liquid gelatin can infiltrate the channel network. If positive pressure is used instead, gelatin infiltrates the interface between the alginate gel and silicone chamber, and also coats the inlets, which prevents the fibrin hydrogel from adhering to the (hydrophobic) silicone chamber. When the gelatin is liquefied, this opens leak paths around the fibrin hydrogel. The alginate gel was chosen to withstand the applied negative pressure without collapsing.

The thermoplastic model has in its surface 12 µm thick terraces due to the layer-by-layer nature of the 3D printing process. This fine stepping is visible in the gelatin template, as shown in figure 2*e*, which demonstrates a high casting fidelity of surface features. On a larger scale however, the overall channel structure is less well preserved due to the weak mechanical properties of gelatin and the suction employed for its infiltration into the alginate channels. Additionally, there is a notable change in the diameter of the gelatin features on variation of the volumes of acetone and CaCl_2_ injected into the alginate channels, both of which produce at least some shrinkage of the alginate gel, acting to reduce the volume of the channels within. The result is channels finer than those modelled in the CAD (figure 2*d*). However, as shown in figure 2*f–m*, there is good correspondence between the 3D printed model and gelatin template.

In this study, both fibrin and collagen hydrogels were used to test the use of the gelatin template with ideal tissue construct materials. Fibrin was easier to handle because at 20 mg ml^–1^, the precursor solution has low viscosity and once thrombin has been added, can be cast quickly using a 1 ml syringe. Conversely, at concentrations of 7.5 mg ml^–1^, collagen gel is much more viscous and requires slow casting using a syringe pump. Collagen precursor solutions gel rapidly at room temperature and so must be kept on ice during this process.

The choice of bulk material was influenced by a need for perfusion and thus a requirement for a tight interface to an external pumping system; thus, silicone inlets were used. It is well known that hydrophobic materials, such as silicone and Teflon, have some protein-binding capacity [32]. In this study, we rely on this adsorption of protein to the surface of silicone rather than the use of other agents to directly bond the gel at the interface. This interface, as shown in figure 2*n*, was tested using fluorescent red 1 µm beads, which showed minimal leaking around the silicone inlet surface when used with fibrin gels. Further, perfusion was tested up to 30 ml h^–1^ (at the inlet) with 10 mg ml^–1^ fibrin gels, which showed no signs of leaking from the chamber.

The use of gelatin as the sacrificial template material has the advantage that it is thermally removed at physiological temperatures. This allows the cell-loaded constructs to be immediately placed in incubator conditions and puts minimal stress on the cells themselves. The use of gelatin enables a wide range of materials to be employed as the final cast, beyond those of thermally cross-linked collagen and enzymatically cross-linked fibrin hydrogels. The template is capable of withstanding a viscous casting material, as shown by 7.5% (w/v) collagen (figure 1*h*), which can be infiltrated without significant displacement of the gelatin template, by way of slow casting with a syringe pump. The release of the template constituents upon thermal dissolution impacts minimally upon the surrounding cellular environment due to the relatively benign nature of gelatin. We postulate that the gelatin template could be used with other hydrogels of relevance to the tissue engineering field, such as alginate, hyaluronic acid, Matrigel, synthetic materials such as PDMS, and also interpenetrating polymeric networks.

**Figure 3.**
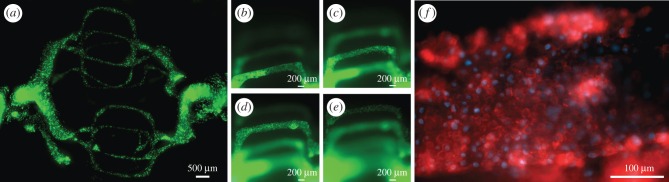
(*a*) HUVECs, labelled with green Cell Tracker dye, were injected into the channels of 20 mg ml^–1^ fibrin hydrogels at 2 × 10^7^ cells ml^−1^ and left overnight, prior to perfusion at 0.1 ml h^–1^. (*b–e*) After 8 days, all four channel layers of the vascular network have high levels of seeded HUVECs. (*f*) CD31 immunostaining was performed on hydrogels containing HUVECs in the channels in order to determine the early formation of tight junctions between endothelial cells. Shown here is a single channel lined with HUVECs, after 8 days of perfusion at 0.1 ml h^–1^. Red and blue colours display cell–cell adhesion marker and nuclear stain, respectively.

As a preliminary evaluation of the vascular system, a series of pilot cell experiments was performed. HUVECs were seeded into the channels via the inlets, as shown in figure 3*a*. HUVECs spread evenly around the symmetrically bifurcating channels and, after several hours, had adhered to the hydrogel channel walls. After 8 days of perfusion, a higher number of HUVECs was observed in all four channel levels of the template (figure 3*b–e*). These cells were perfused at relatively low flow rates (0.1 ml h^–1^) so as to not dislodge the cell layer in the initial stages of the endothelium formation. Further, the observation of high numbers of cells over all layers shows that flow is not confined to a single layer and that nutrients and oxygen would reach a fully 3D volume when cells are added to the bulk. Immunocytochemistry for the cell adhesion marker CD31 showed the formation of tight junctions between HUVECs in the channels (figure 3*f*).

**Figure 4.**
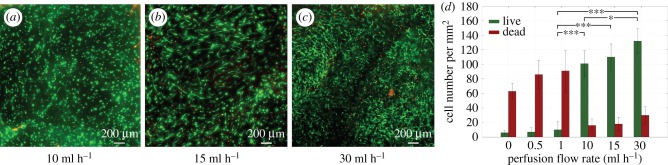
To determine the ability to support cells in thick constructs, HDFs were encapsulated in 10 mg ml^–1^ fibrin gels at 1 × 10^6^ cells ml^−1^ and perfused at a range of flow rates. After 7 days, the gels were cross-sectioned and stained with calcein AM (green) and ethidium homodimer-1 (red). (*a–c*) Live–dead staining for flow rates at which the majority of cells are viable. At 10 ml h^–1^, most cells are round while at 30 ml h^–1^, most cells are spread out. Shown is a cross-section through the middle of the gel normal to the long axis of the channels. (*d*) In order to quantify cell viability at different flow rates, the number of live and dead cells were counted in sample regions. Static conditions (no perfusion) and low flow rates produced constructs with high numbers of dead cells and flow rates above 10 ml h^–1^ produced constructs with high numbers of live cells (*p < 0.05, ***p < 0.001). Comparisons show a statistically significant difference between live cell numbers for flow rates of 1 ml h^–1^ and 10–30 ml h^–1^, and for those between 10 ml h^–1^ and 30 ml h^–1^.

To evaluate the ability of the channel network to support cells in the bulk of the hydrogel, HDFs were mixed into the fibrin precursor solution prior to gelation and cell-loaded fibrin gels (10 mg ml^–1^) were perfused at a range of flow rates (0.5–30 ml h^–1^). After 7 days, a live–dead viability assay was performed by cross-sectioning the gel and staining the central surface with calcein AM and ethidium homodimer-1. There was a notable difference in the viability and cell spreading in the constructs at different flow rates; good viability of HDFs was observed in the bulk for flow rates above 10 ml h^–1^ and further showed increased cell elongation with increasing flow rate, as shown in figure 4*a–c*. To quantify any improvement in viability, live and dead cells were counted in representative sample regions and plotted against flow rate, as shown in figure 4*d*. Static conditions (non-perfused) or low flow rates were incapable of supporting the metabolic requirements of the cells. Flow rates of 10–30 ml h^–1^ were shown to be suitable for maintaining cell viability.

It is conceivable that with improvements in commercial 3D printing technologies, models could be produced with higher precision, smaller feature sizes, and in new materials with improved mechanical, physical, and chemical properties. The process described here, of converting these 3D printed models into gelatin vascular templates, and subsequently cellularized hydrogel materials, would utilize these advances. Further, as we make use of standard 3D printing technology, the fabrication method is relatively inexpensive in comparison with other approaches, such as those requiring custom-made printers, and so could be widely used for 3D network formation in cellularized constructs.

## 4. Conclusion

Current vascularization methods are limited in their capacity for supporting a large construct volume and achieving full three-dimensionality. We report a new method for producing vasculature, which is hierarchical, 3D, and perfusable, in a fully cellularized and bioactive hydrogel. Vascular networks are created by converting 3D printed thermoplastic material into gelatin which, importantly, can be used in cellularized environments cast from extracellular matrix materials and can be removed efficiently for prompt perfusion. We were able to fabricate channels with diameters of 200 µm and apply flow rates comparable with physiological conditions. Further, the ability to use bioactive hydrogel materials with our process enables the support of primary cells, displaying tight cell junctions in the channels, and a high viability and cell spreading in the bulk. These thick constructs are suitable as an inexpensive platform for 3D cell studies and engineering tissue *in vitro*.
